# Neural and perceptual manifolds regarded geometrically

**DOI:** 10.1167/jov.26.10.1

**Published:** 2026-10-01

**Authors:** Gerald Westheimer

**Affiliations:** 1 Department of Neuroscience, University of California, Berkeley, CA, USA

**Keywords:** multiple signals, state of neural system, identity preservation, dimension reduction, untangling

## Abstract

Challenges face the neuroscientist when probing an organism's nervous system yields hundreds or thousands of simultaneous independently recorded signals. One way of dealing with this is to gather all *n* signals into a single point in an *n-*dimensional space, called the *state* of the nervous system. Structures created by the assembly of states formed by a coherent behavioral entity, such as the generalized sensing or perceiving of an object or the performing of a motor action, are called *manifolds.* Geometrical operations involving manifold properties have the potential of characterizing the neural organization underlying observed modules of behavior and predicting expected changes with learning or with the passage along streams of neural processing. Manifold analysis depends critically on the nature of the entered data and the match of the mathematical and computational tools applied to them.

## Introduction

Technological advances have revolutionized neuroscience's ability to learn the representation in the brain when an object is shown to an organism or when a motor task is performed. Single-neuron recordings have given way to the acquisition of signals from many sources; the available description now consists of *n* simultaneously recorded quantities, say, the activity of neurons, *n* being in the hundreds or thousands. As a consequence, the analysis requires an entirely different mindset. The aim is not that of overviewing, aggregating, or summarizing the data as statistics but the opposite: identifying and attempting to tease out inherent structure. The prominent initiative of achieving this through mathematical strategies is reviewed and placed in perspective here: With all the depth and power, the scope and limitations of their tools need to be recognized by all sides.

The first step is a methodology to lay out the data. The impulse rate of a single neuron at any one time is a point along the x-axis. For two simultaneously recorded neurons, it can be visualized as a point in the x,y space with x and y coordinates. Similarly, for *n* neurons, it can be thought of as a point in an *n-*dimensional space, a single event characterized by *n* separate values. In accord with the nomenclature in the physical sciences, it is called the *state* of the system. Aggregates of this *n-*dimensional variable are called *manifolds*, a term Riemann (1854) introduced in his seminal formulation. Over the years, it has accrued many specialized connotations (Scholz, 1999); hence, it will be closely defined on each use.

Different but related events, such as the evolution in time or change in conditions, will make the point (state) occupy different locations, generating an *n-*dimensional structure. Such a set of states described by empirical data over a collection of experimental conditions is called a *data manifold* (Tenenbaum, de Silva & Langford, 2000). If it is made up of states defined by neuronal data that have been recorded or that perhaps are in principle recordable, it is a *neural manifold* (Chung & Abbot, 2021).

Examples of neural manifolds are the ranges of states (the activities in populations of *n* neurons in the visual stream) when the display conditions of an object are changed (size, luminance) or in a population of neurons in the motor system when the animal performs a motor task. Although there have been earlier analyses of population neural activity—for example, the neural “vector” model of Georgopoulos, Kalaska, Caminiti & Massey (1982)—manifold formulation is much richer and has the potential, as we shall see, for very sophisticated analysis.

But at this stage, the manifold is still the description of raw data. The specific kind of measurement on which it is based (e.g., impulse rate in one neuron) must always be kept in mind: Operations, formulations, interpretations, or predictions performed on the manifold pertain only to the restricted sample of the whole neural system from which it was gathered.

More advanced thinking sometimes leads to *Gedankenexperimente*, in which structures in the *n*-dimensional continuum are based not on actual measurement but on wider ranges of possible states that have not (yet) been realized; much discussed are those in which a specified object category is preserved for the observing organism—say, all target configurations identified as a circle or a dog. The resulting structure, the collection of the states (points in the *n*-dimensional aggregate of all possible activity levels) for all identity-preserving objects, is called a *perceptual manifold.*

It should be mentioned parenthetically that, since Riemann's days, mathematicians have actively developed more nuanced and more specific definitions of manifolds. In their most rigorous form, some even deny the neural and perceptual manifolds the label “manifold,” but these arguments can safely be ignored for discussions here. In neuroscience, manifolds begin with the conceptual collapsing into a single point, the state, of *n* different individual measurements, followed by the gathering of a cohering collection of *m* such points: an *m* × *n* matrix thought of as a configuration, composed of *m* points located in an *n*-dimensional space.

This mode of marshaling the findings enables attempts at understanding manifolds’ inherent structure in the interest of such research directions as how perceptual categories are established or how neuronal properties change during learning, or, more generally, finding rules of connectivity and common input. When regarded geometrically, these may take the form of ironing out structural irregularities of manifolds to endow them with desired properties, or of compacting the ensemble size through discovery of covariance (common input) at the recording sites.

The literature now abounds with many admirable studies applying geometrical theory to *n*-dimensional manifolds. This proceeds as theory usually does: Reformulations are advanced based on plausible postulates and their consequences examined for possible realistic and empirically verifiable forms. The wider ramification here lies in the fact that guidelines for induction are sought not only in pure logic or mathematics or in the success story of modern theoretical physics but also in the highly relevant and richly populated arena of behavioral sciences.

One example of this approach is described in a pioneering series of papers whose basic premise is that object discrimination (“dog” and not “cat”) requires the perceptual manifolds of the two categories to be sufficiently separated in the state space that the state generated by a new target presentation clearly falls within one and not the other manifold (Chung & Abbot, 2021; Chung, Lee, & Sompolinsky, 2018), a process called “untangling.”

Since it is inevitable that perceptual manifolds are extended in size, uneven in shape (“like crumpled pieces of paper”), and intertwined, this invites conjectures on what changes in their geometrical properties would make them more separable. Though phrased in mathematical terms at the outset, these changes have to be framed within their biological (brain structure and function) and cognitive contexts. But this is where the dilemma enters. In the original manifolds, the points have the precise meaning of (measured or potentially measurable) activity in actual neurons. Smoothing or debulking manifolds can still pass muster as allowing this interpretation (i.e., reasonable idealization of actual experimental findings). Unlike experiments in which the same set of neurons is studied during, say, learning to examine the effect of contextual or top-down influences, more radical operations of dimension reduction move discussion into the realm of submanifolds. Here it needs new definitions of what the dimensions now stand for.

Though the original manifold may appear small compared with all the neurons in play, fewer degrees of freedom may be involved in accomplishing the task, a sort of abstract that contains a more concise version of its essence. There is the proposition that this is in fact implemented in the progression of remapping and rechanneling of object representation along the ventral visual stream, with the implication that geometrical manifold manipulation can lead to the prediction of neurons’ properties in sequential stations of cortical streams. This is now the province of a dedicated group of computational neuroscientists whose thinking is informed by a heady mix of empirical data and a wealth of tools assembled from mathematics, theoretical physics, and data science.

The connection may be noted here between perceptual manifolds and Felix Klein's formulation of geometry as the study of structures that remain invariant under transformation groups (Klein, 1893). Postulating category preservation under a defined range of presentations is tantamount to identifying the manifolds and rules of transformation as a Group in the group-theoretical meaning. From there, it is but a step to Ernst Cassirer's long-dormant anticipation expressed in the title of his “The Concept of Group and the Theory of Perception” (Cassirer, 1944).

So far, manifold has been used in the meaning given by Riemann in 1854: the *n-*dimensional expanse of all possible neural states or, in the diminished sense of current computational neuroscience, the structure formed by a set of neural states that satisfy defined criteria. It has, however, a history even more venerable than Riemann's celebrated *Habilitationsschrift.* Laying out the taxonomy of steps, stages, and entities that he thought led to an aware observer's perception and cognition of a real object, Kant (1781), in his 1781 *Critique of Pure Reason*, gave a central place to *manifolds* (*Mannigfaltigheiten in der Anschauung*). They are constructs in the mental world synthesized from sensory impression, have time and space imprints, have been subject to formatting influences from internal sources, but have yet to acquire perceptual identification. As a real object's first internal representation, Kantian manifolds’ resemblance to the manifolds of computational neuroscience merits examination.

But they live in the mental world, whereas, as they appear in the literature (DiCarlo & Cox, 2007), neural manifolds are assemblies of measured neural states, quantities observed in real organisms presented with real objects. Kant would accept their existence but would insist on their being in a separate realm and knowable to the mental only indirectly.

This separation is not so compelling when computational neuroscience proceeds to the next level, where the substance of manifolds is no longer actual observables (or their easily envisaged extensions, such as a sequence of small changes in object attributes) but identity-preserving invariances, the full panoply of pattern displays that, for the observer, remain in the same category. And to permit category discrimination, these perceptual manifolds need to undergo sophisticated operations based on mathematical, in particular geometrical, thinking that have valid claims to belong to the mental rather than material world.

Hence, we are faced with the dialectic that Fechner (1860) confronted. His aim was to establish a “functional relationship between the corporal and mental worlds,” and he presciently distinguished *outer* and *inner* psychophysics, depending on whether the mental world's interface with the material world was the body's soma or the nervous system. In his time, only an outer psychophysics was possible, and it proved to be an immensely productive enterprise, providing the platform for the discovery of a wide range of capabilities, the neural substrates of many of which remain to be elucidated. One hundred and fifty years after he foreshadowed it, Fechner's inner psychophysics is taking the form of perceptual manifolds, involving neural recording, mathematical operations, and data from perception and cognition.

The utilization of the manifold as an ensemble of neural states, however, need not be handicapped by agonizing over how to reach the mental world. Its essence includes sets of multiple measurements, obtained in a cluster of related events, situations, or configurations. To correctly assign a presented object to a category, overlapping manifolds need untangling and separating. For Kant, this process involves synthesizing an *Object in Perception* from a *Manifold in Anschauung*, and takes place in the mental world. For present and future computational neuroscientists, on the other hand, studying the evolution of neural manifolds can be based entirely on physical observables. True, the tracing of changes would face severe difficulties where manifolds evolve through remapping by passing forward along cortical streams. But the task would become much more manageable where changes can be observed in the same large set of neurons (“untangling” in situ), as top-down and local contextual influences are modifying neural activity in the cause of category distinction (perceptual learning). There is great promise for the productive interplay of data acquisition from the intact behaving organism and advanced geometrical thinking.

A final caveat. There is no better exemplar of the nexus between empirical and computational neuroscience than a manifold. Because everything depends on them, the data from which manifolds are generated merit the fullest description and closest examination. And because the value of a manifold is the opportunity it affords for deployment of advanced mathematical and computational models, it obliges theoreticians to ensure a genuine match of their concepts to the nature of the neural data and the intent and meaning of the inquiry. Hence, even as manifold analysis elevated neuroscience to a higher level, it places heavier demands and calls for a deeper knowledge base in all who would engage in it.
